# The experience of providing young people attending general practice with an online risk assessment tool to assess their own sexual health risk

**DOI:** 10.1186/1471-2334-9-29

**Published:** 2009-03-12

**Authors:** Jade E Bilardi, Lena A Sanci, Christopher K Fairley, Jane S Hocking, Danielle Mazza, Dot J Henning, Susan M Sawyer, Michelle J Wills, Debra A Wilson, Marcus Y Chen

**Affiliations:** 1 Melbourne School of Population Health, The University of Melbourne, Carlton, Victoria 3053, Australia; 2 Department of General Practice, The University of Melbourne, 200 Berkeley Street, Carlton, Victoria 3053, Australia; 3 Melbourne Sexual Health Centre, 580 Swanston Street, Carlton, Victoria 3053, Australia; 4 Centre for Adolescent Health, Royal Children's Hospital, 2 Gatehouse St, Parkville, Victoria 3052, Australia; 5 Department of General Practice, Monash University, Building 1, 270 Ferntree Gully Rd, Notting Hill, Victoria 3168, Australia; 6 Department of Paediatrics, The University of Melbourne, Royal Children's Hospital, Flemington Road, Parkville, Victoria 3052, Australia; 7 Key Centre for Women's Health in Society, School of Population Health, The University of Melbourne, Level 2, 723 Swanston Street, Carlton, Victoria 3010, Australia; 8 General Practice Divisions Victoria, 458 Swanston Street, Carlton, Victoria 3053, Australia; 9 Young Peoples Health Service, 19 King Street, Melbourne, Victoria 3000, Australia; 10 Dianella Community Health Inc, 35 Johnstone Street, Broadmeadows, Victoria 3047, Australia

## Abstract

**Background:**

Targeted chlamydia screening has been advocated to reduce chlamydia associated reproductive sequelae. General practitioners are well positioned to play a major role in chlamydia control. The primary aim of this pilot study was to measure the effect of offering an online sexual health assessment tool, *Youth Check Your Risk*, on chlamydia testing rates among young people attending general practices. The secondary aim was to test the acceptability of the tool among general practitioners and young people.

**Methods:**

General practitioners at three practices in Melbourne, Australia, referred patients aged 16 to 24 years to *Youth Check Your Risk *http://www.checkyourrisk.org.au for use post-consultation between March to October 2007. The proportion of young people tested for chlamydia before and during the implementation of the tool was compared. Acceptability was assessed through a structured interviewer-administered questionnaire with general practitioners, and anonymous online data provided by *Youth Check Your Risk *users.

**Results:**

The intervention did not result in any significant increases in the proportion of 16 to 24 year old males (2.7% to 3.0%) or females (6.3% to 6.4%) tested for chlamydia. A small increase in the proportion of 16 to 19 year old females tested was seen (4.1% to 7.2%). Of the 2997 patients seen during the intervention phase, 871 (29.1%) were referred to *Youth Check Your Risk *and 120 used it (13.8%). Major reasons for low referral rates reported by practitioners included lack of time, discomfort with raising the issue of testing, and difficulty in remembering to refer patients.

**Conclusion:**

Offering an online sexual risk assessment tool in general practice did not significantly increase the proportion of young people tested for chlamydia, with GPs identifying a number of barriers to referring young people to *Youth Check Your Risk*. Future interventions aimed at increasing chlamydia screening in general practice with the aid of an online risk assessment tool need to identify and overcome barriers to testing.

## Background

*Chlamydia trachomatis *is the most common bacterial sexually transmitted infection (STI) worldwide [1]. Screening programs and widespread testing have been implemented in a number of countries as a means of improving chlamydia control, with varying success. In Australia, chlamydia notification rates have risen nearly fourfold over the past 10 years (1999–2008) [2] prompting the Federal Government to recently fund various initiatives aimed at chlamydia control, including a pilot chlamydia testing program in general practice.

Even in countries where national screening programs have been implemented, sustained reductions in chlamydia prevalence have not been achieved [3]. To achieve a sustained reduction in prevalence, it is likely higher rates of screening than have been seen are required [4]. Interventions aimed at both general practitioners (GP) and patients that effectively increase chlamydia screening rates in primary care settings are needed [3,5].

In Australia, GPs currently see nearly 90% of women and 70% of men aged 15 to 24 years at least once a year, providing an ideal opportunity for targeted chlamydia screening [3]. However, barriers to opportunistic screening include: lack of time; limited knowledge of testing methods, screening guidelines and the benefits of screening; discomfort raising the issue of testing and taking sexual histories; and a perception that patients are reluctant or embarrassed to discuss sexual health issues [6-11]. Furthermore, young women report reluctance to discuss their sexual history during the consultation as a major barrier to chlamydia screening [12].

To help overcome these barriers, the Melbourne Sexual Health Centre (MSHC) implemented an online self-completion program entitled Check Your Risk (CYR) to allow individuals to obtain an assessment of their sexual risk and recommendations for STI tests that they could take to their GP. The recommendations were based on answers online users provided to a series of questions relating to their recent sexual activity [13]. The recommendations were downloadable in the form of a letter to the GP and contained technical advice for GPs on appropriate STI screening tests. Potential users of CYR were informed that their responses are confidential and that no personally identifying information was required. CYR was used by individuals at substantial risk for STIs [13].

The primary aim of this study was to determine whether offering information about and access to a youth version of the online self-completion tool *Youth Check Your Risk *(YCYR) in general practice would lead to increased chlamydia screening of young people attending their GP for any reason. The secondary aim was to test the acceptability of the tool among GPs and young people.

## Methods

### Participants

Three general practices in metropolitan Melbourne, Australia, were recruited from a database of over 100 research interested practices listed by the Department of General Practice at The University of Melbourne. Practices were eligible if they saw a minimum of 25 young people aged 16 to 24 years each week, had no current chlamydia screening program in place and preferably had a private area where a computer could be set up for patients to utilise YCYR. Approximately 70 practices were telephoned in order to recruit three eligible practices who were interested in participating after receiving written and verbal information about the project. Practices were each given an honorarium amount of $1,000 (AUD) in acknowledgement for GPs' time spent out of usual roles in participating in this study.

### Intervention

A focus group was held with 13 young people (16 to 24 years, Male: female, 4:9) and another with five practicing GPs (Male: female, 1:4) linked with The University of Melbourne to inform how the original online CYR tool could be modified to specifically focus on sexual risk screening of young people in primary care. Modifications included the re-design of the website using 'youth friendly' website graphics, additional questions on condom and contraceptive use, and a section for users to provide feedback on the site. The YCYR recommended chlamydia screening to all males and females aged 16 to 24 years who reported any sexual partners in the previous 12 months http://www.checkyourrisk.org.au.

Participating GPs were provided with a printed educational package that covered the management and treatment of chlamydia, including the Royal Australian College of General Practitioner guidelines on chlamydia screening [14] and a free call telephone number for GPs wanting advice from a sexual health physician. Anecdotal feedback from focus group GPs and general pre-trial discussions with participating GPs indicated that they wanted any educational component kept to a minimum given their workloads and time limitations.

**Figure 1:**
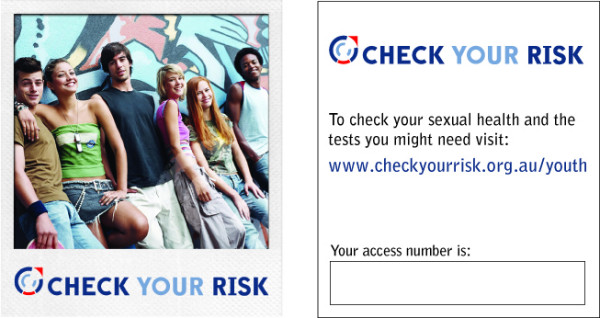
**Youth Check Your Risk Information Card**. Information card used to refer young general practice patients to *Youth Check Your Risk*.

GPs were then asked to refer all people aged 16 to 24 years to the YCYR website, regardless of their reason for presentation, by giving them an information card containing the web address (Figure 1). Cards were labelled with a unique access number which patients used to log into the site, ensuring that only patients in the study could access the site and that the number of users from each practice could be identified. Cards and posters advertising the site were also available in the waiting rooms and practice reception areas. Two of the practices were able to provide a private location where young people had the option of accessing the YCYR website on a computer within the practice. One location was a small room used as a sick bay area, located between reception and consulting rooms; the other was in a large room behind reception, designated as a 'staff only' area, where the computer was positioned in a corner to ensure viewing of the screen by users only.

Further informational leaflets were provided to practices to place in their reception areas at approximately the three month mark of the trial, when it was evident that lower than expected numbers of young people were accessing the site. GPs were also sent a reminder letter of their participation in the study.

### Outcome measures

The proportion of young people tested for chlamydia in the six months before (September 2006 to February 2007) and the six months of the intervention (March to October 2007) was calculated using data on chlamydia tests ordered by the practices from the pathology laboratories servicing each practice. Data on the number of visits by patients in the target age group were obtained from each practice's electronic medical records databases.

A structured, audio-taped, interviewer-administered questionnaire with some open ended questions was used to gauge GPs' views on the importance of chlamydia testing, the acceptability of the intervention, and the barriers they saw to chlamydia screening in general practice. Young people's views on the acceptability of being referred to YCYR by their GP and using YCYR were sought through two means. Firstly, via the feedback page on YCYR which asked a series of questions, including where they accessed the site from, whether they found the website useful (7 point scale), and how likely it was that they would consult a GP with the STI recommendations (7 point scale). A free text space was available for comments or suggestions. The second means of obtaining young people's views was through invitation on the website's feedback page and on waiting room posters to phone a 1800 toll free number to participate in a brief structured interview. Young people were offered two free movie tickets for taking part in this interview. Further advertisement of the free movie tickets was placed on informational leaflets and posters three months into the trial when no young people had contacted the researchers to participate in an interview.

### Analysis

The proportion of young people tested for chlamydia in the six months before and the six months of the intervention, was compared for each practice using a test for equality in proportions. The difference in the proportion and 95% confidence intervals were calculated using exact methods for binomial proportions and a two-sided p value was presented. Descriptive analysis was conducted on quantitative GP interview data and anonymous online data provided by users. Answers to open ended questions were transcribed and analysed by JEB and LAS thematically using N-Vivo 7.

Ethical approval for this study was granted by the human research ethics committee of The University of Melbourne.

## Results

Fourteen GPs across the three practices participated in the study: six were male and eight female. Four GPs were aged 31 to 40 years, four 41 to 50 years and six 51 to 60 years. Of the three participating practices, two were university affiliated and saw a high number of young people in the age group targeted for this intervention.

Over the study period, 2997 patients aged 16 to 24 attended the three clinics, of which 2002 (66.8%) were female and 995 (33.2%) male. Of these, 871 (29.1%) patients were referred to YCYR either by a GP, reception staff or through collecting a YCYR card in the waiting room. Of these 871 patients, 120 (13.8%) used YCYR.

The proportions of patients aged 16 to 24 who were tested for chlamydia before and during the implementation of YCYR are shown in Table 1. The chlamydia prevalence in this group among females (n = 5/129) was 3.9% (95% CI: 1.4, 9.3) and among males (n = 3/30) was 10.0% (95% CI: 2.6, 27.7). In the six months prior to the implementation of YCYR the chlamydia prevalence among females (n = 5/98) was 5.1% (95% CI: 1.9, 12.1) and among males (n = 3/20) was 15.0% (95% CI: 4.0, 38.9).

**Table 1:** Proportion tested, by age and sex, before and during YCYR*

Sex & Age Group	Number tested	Number not tested	% tested	% diff (95%CI)	*p-value
Male 16–19					
Before	4	216	1.8	1.1 (-1.3, 3.5)	*p *= 0.45
During	2	289	0.7		
Male 20–24					
Before	16	516	3.0	1.0 (-1.3, 3.2)	*p *= 0.46
During	28	676	4.0		
Total males					
Before	20	732	2.7	0.4 (-1.3, 2.0)	*p *= 0.77
During	30	965	3.0		
Female 16–19					
Before	13	305	4.1	3.0 (-0.3, 6.4)	*p *= 0.10
During	37	483	7.2		
Female 20–24					
Before	85	1145	6.9	0.7 (-1.3, 2.7)	*p *= 0.83
During	92	1390	6.2		
Total females					
Before	98	1450	6.3	0.1 (-1.6, 1.8)	*p *= 0.95
During	129	1873	6.4		

*The proportion of young general practice patients who were tested for chlamydia in the 6 months before and 6 months of *Youth Check Your Risk*

### YCYR Users

The mean age of YCYR users was 21 years. Data on reported sexual practices from those who accessed YCYR are shown in Table 2. Of the 120 young people who used YCYR, 14 completed the online feedback page. Users gave the site a mean rating of 4.8 (1 = not at all useful, 7 = very useful) when asked how useful they found the site and a mean score of 5.2 (1 = not at all likely, 7 = very likely) when asked how likely it was that they would consult a GP with the STI recommendations. Of the 14 respondents, only two accessed the site from the general practice computer. No young people contacted researchers to participate in an interview.

**Table 2:** Information provided by users in completing YCYR, by sex and collectively

	Male	Female	Total	
CYR Questions	Number		Number	%
Gender	56	64	120	100.0%
MSM or WSM	5	3	5	7.7%
MSW or WSM	51	61	112	93.3%
Born in Australia	31	45	76	63.3%
Sex with new partner > 12 months	40	35	75 (*115)	65.2%
Sex with new partner from overseas > 12 months	22	5	27	23.5%
Sex with someone with STI > 12 months	1	1	2	1.6%
Have injected drugs not prescribed by a GP	1	2	3	2.5%
Do not use condoms 100% of the time	31 (*53)	47 (*63)	78	67.2%
Reported having sex with a new casual partner while also reported not using condoms 100% of the time	23 (*53)	24	47 (*115)	40.5%

MSW – men reporting sex with women only

MSM – men reporting sex with any men

WSM – women reporting sex with men only

WSW – women reporting sex with any women.

* Number of eligible responses – Due to the YCYR decision trees, not all users were required to answer all questions within the program accounting for varying response numbers

### GP feedback

Of the 14 GPs involved in the study, 10 participated in the structured interviewer-administered questionnaire at the completion of the study. GPs rated testing for chlamydia as very important (mean 4.5, 1 = not at all important, 5 = extremely important) but only had an average rating on level of interest in the study of 3.5 (1 = not at all interested, 5 = very interested).

The general consensus from GPs was that the YCYR website was clear, easy to follow and a useful way of providing information to users and raising sexual health awareness. The three main barriers identified by GPs in referring patients to YCYR were:

1) Lack of time during consultations, particularly in view of the explanation they felt was inevitably required when referring patients to YCYR and the subsequent questions on sexual health issues that often followed. As one GP noted:

...yes we all know that it's really important to do it [chlamydia testing] but in the end we've got 15 minutes and I spend longer and I'm always running late, but still it's that time issue.

2) Discomfort raising the issue of sexual health, particularly where patients had not presented for sexual health reasons.

3) Difficulty in remembering to refer patients in the target age group.

To bypass these issues, the majority of GPs at one clinic reported leaving referral to YCYR to reception staff.

On the whole, GPs reported that they did not hand out the referral cards or offer chlamydia screening opportunistically to all young patients, but did so when young people presented with high risk behaviours or issues related to sexual health, such as cervical screening, screening for sexually transmitted infections, contraception or genital symptoms. The majority of GPs reported that they did not feel that their testing practices had changed as a result of YCYR; however, participation in the study did raise their awareness and reminded them of the need to opportunistically screen young people for chlamydia.

GPs reported mixed reactions from patients they referred to YCYR, from '*really negative' *and '*indifferent*' to '*fairly responsive' *and '*interested*'. Of the GPs interviewed, only two encountered patients who provided feedback on YCYR. One patient provided general feedback on the service to their GP at a later consultation, while another patient returned to their GP with the printed recommendations. Practices indicated that patients seemed reluctant to use YCYR on site, particularly in the practice in which the computer was set up in the 'staff only' area.

## Discussion

We found that opportunistically offering an online sexual risk assessment tool in general practice to young people did not significantly increase the proportion of young people tested for chlamydia, although a small increase in the proportion of 16 to 19 year old women tested was seen. While GPs generally had a favourable view of the tool, a number of barriers prevented them from referring young people to YCYR and consequently, less than a third of young patients were referred to the site, and of these, few accessed it.

The provision of onsite computers by practices did not seem to improve access by young people, and while no young people provided feedback about this, the location of the computers could have been a deterrent along with the fact that the only reason patients would access them was to use YCYR, hence potentially leaving young people feeling exposed or embarrassed. At a system level, practices may not be equipped to provide patients with a private area in which they can confidentially source online resources.

This study has some limitations. First, we do not know if the small increase in chlamydia testing among younger women was as a direct result of offering YCYR through general practice. In order to protect the confidentiality of patients accessing the website, it was not possible to track health service use of individual patients accessing the site, therefore we do not know how many of the patients who accessed the website actually returned to a GP for chlamydia testing. Nor can we determine which chlamydia tests were the result of the patient accessing the website and being exposed to the recommendations for chlamydia screening. It is possible that testing increased because of a greater awareness of chlamydia screening due to the practices' involvement in the study or because of exposure to the educational package provided to GPs as part of the intervention, rather than as a direct result of referral to YCYR. Secondly, although their views were sought, only a small number of YCYR users provided online feedback and none provided detailed interview feedback despite the incentive offered, so we do not know how most young patients felt about being referred to the website, its on-site access, use of the site or whether they followed up on the advice. Thirdly, as two of the practices were affiliated with universities and serviced high numbers of young people and international students, it is uncertain how generalizable the results are to other practice populations.

Reviews of interventions aimed at change in primary care show that while no intervention is effective in all circumstances, systematically developed, multi faceted interventions tailored to and engaging the target group and addressing barriers and facilitators to change are more likely to be effective [15-17]. A recent review by Ginige et al [5] specifically examining interventions aimed to increase chlamydia screening in primary care found that potentially effective strategies included enhancing GPs' communication skills, particularly around sexual history taking, and increasing GPs' knowledge and awareness around chlamydia, its associated complications, screening guidelines and non-invasive testing techniques. In addition, interventions that offer a combination of educational strategies, including interactive activities – as opposed to printed material only – are more likely to induce greater physician behavioural change [15].

It is possible that a more rigorous methodological approach in this study may have better addressed the existing barriers. In saying this, in a study by Merritt et al [9], despite the use of a simple screening protocol and extensively engaging and informing GPs in a multi-faceted, practice tailored intervention, increases in opportunistic chlamydia screening of young people were at most only moderate and were not sustained, with GPs reporting the same three barriers to testing.

While engaging and increasing GPs knowledge around chlamydia testing is likely to go some way towards facilitating chlamydia screening, it is unlikely to increase opportunistic screening to the levels required to have an impact on chlamydia transmission in the population. Enhancing GPs communication skills, particularly around sexual history taking, may also help, however, as previous research has shown, GPs often do not feel comfortable raising the issue of sexual health during unrelated consultations [8,9] and are less likely to offer screening to patients who are asymptomatic or considered low risk [6].

Furthermore, GPs' perceptions that patient's are embarrassed discussing their sexual health is associated with a reduced likelihood of taking a sexual history and thus assessing a patient's risk [11]. Recent work [12] examining women's attitudes to the introduction of chlamydia screening in general practice has shown that women did not want to be asked a sexual history when being asked to have a chlamydia test. Instead, they wanted to be offered testing based on age, rather than on GPs' assessment of their sexual risk. It could be argued that not taking a sexual history does not constitute best medical practice, however, given the high incidence of asymptomatic chlamydia cases, GPs' tendency towards screening only in high risk or symptomatic cases, and evidence that neither patients nor GPs feel comfortable with sexual history taking, further consideration needs to be given to routinely offering screening to young people according to their age rather than sexual risk. Testing is also more likely to be accepted by young women if it is normalised through wider community education campaigns that highlight the health benefits and destigmatise screening [12]. The recent introduction of human papillomavirus (HPV) immunisation in Australian schools based on age rather than risk profile is an example of the success of this approach [18]. Given the difficulties faced by GPs in remembering to refer patients for testing, further consideration should also be given to investigating whether alerts, programmed into electronic patient management systems, could prove successful in reminding GPs to screen patients presenting in the target age group. Following the success of the reimbursement programs for GPs to increase childhood immunisation rates in Australia [3], it may also be worth investigating whether a similar reimbursement program could prove equally as successful in increasing chlamydia screening rates.

## Conclusion

Offering an online sexual risk assessment tool in general practice did not significantly increase the proportion of young people tested for chlamydia, with GPs identifying a number of barriers in referring young people to YCYR.

It is possible that the intervention required more rigorous development to overcome these barriers. However, before the intervention can be considered ineffective, the way in which it was implemented must also be considered [19]. The low use of YCYR is likely to have been affected by the low number of young people referred to the site by GPs, who reported more often referring patients only when they presented with high risk behaviours, or as was the case with at least one practice, leaving referral to reception staff. A different outcome may have resulted had all eligible patients been referred opportunistically by GPs as intended.

The use of YCYR in general practice therefore should not be discounted altogether. However, given the issues and barriers evident in this study and reflected in other current research, the development of any future interventions, including YCYR, must first address these barriers if they are to effectively increase screening in general practice.

## Abbreviations

CYR: Check Your Risk; GP: General Practitioner; HPV: Human papillomavirus; MSHC: Melbourne Sexual Health Centre; STI: Sexually transmitted infection; YCYR: Youth Check Your Risk.

## Competing interests

The authors declare that they have no competing interests.

## Authors' contributions

JEB was responsible for data collection, analysis and interpretation, and prepared the first draft of the manuscript. LAS contributed to the conception and design of the study, interpretation of data and writing of manuscript. CKF contributed to the conception and design of the study, interpretation of data and writing of manuscript. JSH was involved in study design, assisted in the statistical analysis and critically reviewed the manuscript. DM was involved in study design and critically reviewed the manuscript. DJH was involved in study design, assisted in youth focus group recruitment and critically reviewed the manuscript. SMS was involved in study design and critically reviewed the manuscript. MJW was involved in study design and critically reviewed the manuscript. DAW was involved in study design, assisted in the GP reference group recruitment and critically reviewed the manuscript. MYC contributed to the conception and design of the study, interpretation of data and writing of manuscript

All authors read and approved the final manuscript.

## Pre-publication history

The pre-publication history for this paper can be accessed here:

http://www.biomedcentral.com/1471-2334/9/29/prepub
